# Insights to Human γD-Crystallin Unfolding by NMR Spectroscopy and Molecular Dynamics Simulations

**DOI:** 10.3390/ijms23031591

**Published:** 2022-01-29

**Authors:** Shu-Shun Hsueh, S.-S. (Steven) Wang, Shu-Han Chen, Chia-Lin Wang, W. (Josephine) Wu, Ta-Hsien Lin

**Affiliations:** 1 Department of Chemical Engineering, National Taiwan University, Taipei 10617, Taiwan; naluwan8220@gmail.com (S.-S.H.); sswang@ntu.edu.tw (S.-S.W.); b06504117@ntu.edu.tw (S.-H.C.); 2 Laboratory of Nuclear Magnetic Resonance, Medical Research Department, Taipei Veterans General Hospital, Taipei 11217, Taiwan; clwang5@vghtpe.gov.tw; 3 Department of Optometry, Yuanpei University of Medical Technology, Hsinchu City 30015, Taiwan; 4 Institute of Biochemistry and Molecular Biology, National Yang Ming Chiao Tung University, Taipei 11221, Taiwan

**Keywords:** human γD-crystallin, stability, unfolding, aggregation, cataract, NMR spectroscopy, molecular dynamics simulations

## Abstract

Human γD-crystallin (HGDC) is an abundant lens protein residing in the nucleus of the human lens. Aggregation of this and other structural proteins within the lens leads to the development of cataract. Much has been explored on the stability and aggregation of HGDC and where detailed investigation at the atomic resolution was needed, the X-ray structure was used as an initial starting conformer for molecular modeling. In this study, we implemented NMR-solution HGDC structures as starting conformers for molecular dynamics simulations to provide the missing pieces of the puzzle on the very early stages of HGDC unfolding leading up to the domain swap theories proposed by past studies. The high-resolution details of the conformational dynamics also revealed additional insights to possible early intervention for cataractogenesis.

## 1. Introduction

**Figure 1:**
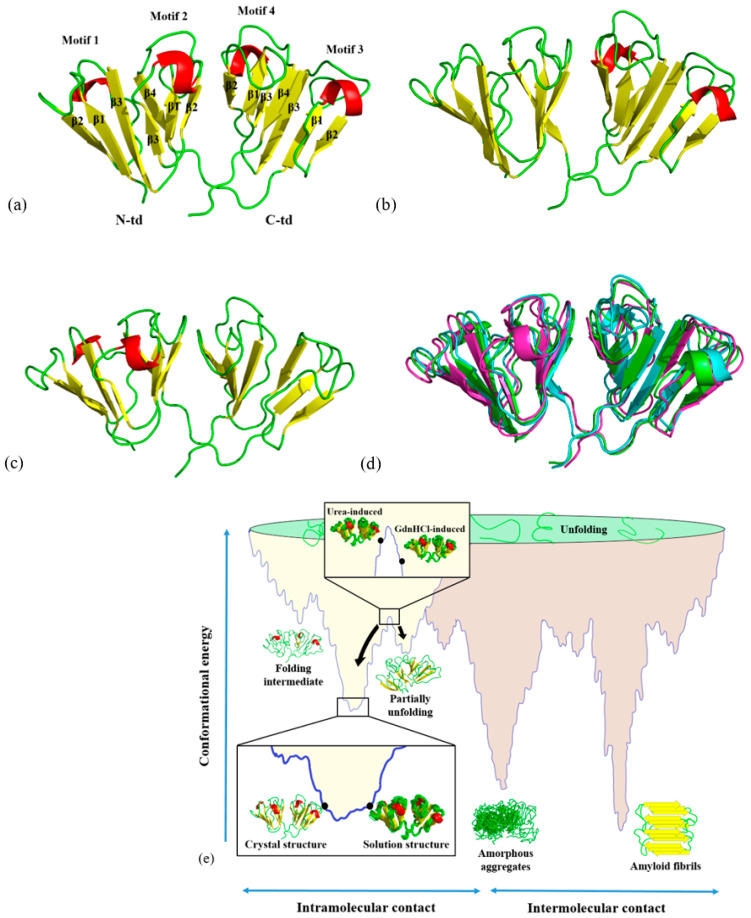
NMR solution structures of HGDC (**a**) without denaturant, (**b**) with 5.0 M urea, and (**c**) with 1.0 M GdnHCl. (**d**) Superimposition of the solution structures of HGDC with and without denaturants. The solution structures of HGDC without denaturant, with 5.0 M urea, and with 1.0 M GdnHCl were presented in green, cyan, and magenta, respectively. (**e**) A hypothetical conformational potential energy surface showing relative positions of the different HGDC conformational states. This schematic diagram is adapted from Fig. 1 of Jahn and Radford (2005) [8].

Proteins, in their native forms, are essential biological macromolecules taking on various biological roles in physiochemical processes. They can also play a structural role by simply being in a natively well-defined folded state to retain order in the body and achieve specialized function, such as maintaining clear vision. One such protein is human γD-crystallin (HGDC), a predominant protein of the eye lens nucleus. HGDC, the second most abundant protein of the lens nucleus [1,2,3] and the most abundant γ-crystallin in the human lens [4], is a 173-residue (with 14 tyrosines, 4 tryptophans, and 6 phenylalanines) globular structural protein with a molecular weight of approximately 20 kDa. HGDC exists as a two-domain protein, where each domain contains two Greek key motifs, composed of intercalated anti-parallel β-strands in each motif (Figure 1a). These four Greek keys are structurally homologous, yet non-identical. The N-terminal (N-td) and C-terminal (C-td) domains are joined by a compact hydrophobic interface, which is composed of a cluster of six hydrophobic residues and two pairs of polar peripheral residues flanking the hydrophobic cluster, resulting in a globular protein about 5 nm in diameter. Evidence pointed out that the interdomain interface plays an important role in maintaining the stability of γ-crystallins [5]. Previous studies suggest that the highly stable structures of crystallins are resistant to damage during the lifetime of the host organism [6,7]. This is an important property for structural proteins, such as the HGDC, as they are retained within the crystalline lens throughout the lifetime of the host and having a highly stable structure is essential to their function for maintaining lens clarity.

The transitioning of native to aberrant, misfolded proteins that leads to massive protein aggregation as the underlying basis for protein aggregation diseases, such as cataract, has long been a mystery that baffled the minds of biophysicists for decades. In order to understand this change from functional to disease-induced protein, a fundamental method is to probe into the molecular detail of the unfolding/refolding dynamic pathways of the key protein involved. One way to do so is by the use of chemical denaturation, such as urea and guanidine hydrochloride (GdnHCl), to explore the partially unfolded protein intermediates. By doing so, one may be able to identify the general factors or mechanism(s) that cause proteins to destabilize [9,10,11,12,13], paving the way down the eventual disease pathway. Chemical denaturants are known to disrupt the non-covalent interactions that stabilize the native protein structure and, by doing so, allow one to search for universal nature of the structural changes in a particular protein during the process of unfolding, misfolding, and aggregation involved in the disease pathology.

In this study, we combined solution-NMR experiments and molecular modeling to probe for the conformational changes in HGDC that may give us insights to the disease pathway of cataract formation. Three solution-NMR structures, native and high energy state structures obtained from denaturing solutions (urea and GdnHCl), were submitted for molecular dynamics (MD) simulations in a water solvent to understand the structural changes these proteins may undertake during the process of refolding/misfolding when removed from the denaturing environment. Traditionally, HGDC simulations have been performed in either high temperature and/or urea denaturant using the native structure derived from X-ray crystallography experiments [14,15,16,17]. However, the rigid crystal structure may not account for the natural dynamic state of the protein and paint a realistic picture of the conformational changes that can occur. Our study is the first to use solution-state NMR structures of HGDC as starting structures to compare and contrast the dynamics of the structural changes arising from different co-solvent environments.

## 2. Results

### 2.1. NMR Solution Structures of HGDC in the Absence and Presence of Denaturants

In the previous studies [17,18], we have predicted the region of HGDC that may be involved in its aggregation process under low-pH conditions by MD simulations using the X-ray crystal structure of HGDC as a starting structure. In the present study, we applied a similar approach to predict the unstable regions of HGDC and to examine the sequence of initial unfolding events that may lead to misfolding, and ultimately result in HGDC aggregation. We first determined the solution structures in the absence and presence of denaturants (5.0 M urea and 1.0 M GdnHCl) using NMR spectroscopy. The two-dimensional ^1^H-^15^N-HSQC spectra of HGDC in the absence and presence of denaturants with the assigned residues are indicated in Figure S1 (Supplementary Materials), while the experimentally measured ^1^D_N-HN_ RDCs of HGDC in the absence and presence of denaturants are shown in Figure S2 (Supplementary Materials). We derived the three-dimensional solution structures of HGDC based on these NMR data. Figure 1a–c shows the three-dimensional structures of HGDC from buffer, 5.0 M urea, and 1.0 M GdnHCl solutions, respectively. An overlay of these three solution structures is shown in Figure 1d. Although the overall three-dimensional folds of the three HGDC structures are similar, they are not completely the same. Figure 1e shows the positions of the HGDC structures in respect to a hypothetical conformational potential energy surface [8]. Different solution structures of HGDC can be seen positioning at different levels on its conformational potential energy surface. For example, the structure in buffer solution is positioned at the lowest point of the energy surface, while the other two denaturant-induced higher energy state structures are positioned at higher levels. For simplicity, in the subsequent sections, denaturant-induced higher energy state structures are referred to as urea-induced structure and GdnHCl-induced structure, while the solution structure in buffer is simply solution structure.

We used these NMR structures as the starting conformations for molecular dynamics (MD) simulations to understand which paths (unfolding, misfolding, or refolding) these protein structures may take when simulated in physiological solution under high temperature. By doing so, we were able to gain more insights into the relative unstable regions of the HGDC and understand how they destabilize during the initial unfolding process. Knowledge gained from this study may aid in the design of aggregation inhibitors to treat cataract.

### 2.2. Relative Conformational Changes between the HGDC Structures Simulated in 343K

**Figure 2:**
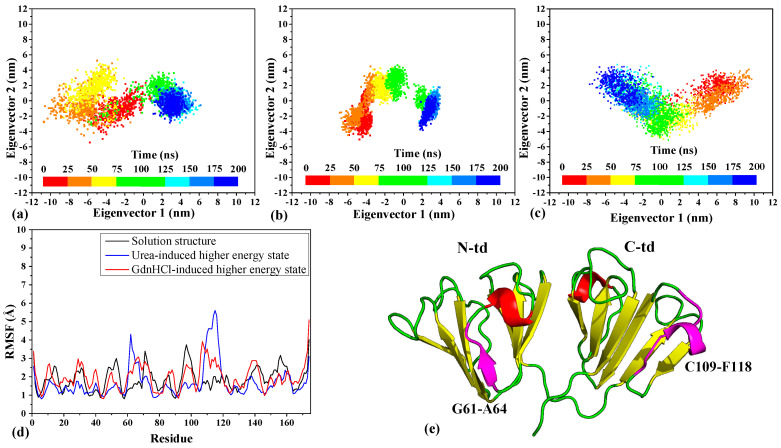
The 2D scatter plots, root–mean–square (RMSF) per residue for the simulated trajectories of HGDC structures in physiological solution (343 K) for 200 ns, and the secondary structural schematic of HGDC. The scatter plots are projected to the top two principal components for the simulated trajectories of (**a**) solution, (**b**) urea-induced, and (**c**) GdnHCl-induced structures. (**d**) Backbone RMSF values for all three HGDC structures. (**e**) Residues G61–A64 and C109–F118 represented in magenta highlights in the HGDC structure.

We first performed 200-ns MD simulations in 343 K to see how the denaturant-induced higher energy structures would change when placed back in physiological solvent environment. The destabilization effects of the denaturants can be seen in the RMSD analysis of the MD simulation trajectories (Figure S3, Supplementary Materials). The two denaturant-induced structures yielded higher RMSD values with greater fluctuations seen in the GdnHCl-induced structure. In comparison with the solution structure (with the RMSD values leveled off below 3.0 Å), both the urea- and GdnHCl-induced structures resulted in higher RMSD values with the final values leveling off just below 4.0 Å for the urea structure and fluctuating between 4.5~5.0 Å for the GdnHCl structure. Based on the PCA analysis of the essential dynamics [19] in the simulated trajectories, we found that the ensemble of conformations for the solution structure is mainly scattered for the first 100 ns with a more distinct cluster pattern forming thereafter (Figure 2a), which corresponds to the equilibration of the structure around this time seen in the RMSD analysis (Figure S3, Supplementary Materials). Similarly, but more definitively, a cluster break can be seen in the urea-induced ensemble around the same time (Figure 2b), also corresponding to the plateauing of the RMSD values. In contrast to the distinctive clustering of the solution and the urea-induced structures during the simulations, the ensemble of GdnHCl-induced conformations remained largely scattered (Figure 2c). Although we see a bifurcation in the scatter around 50 ns, no distinctive cluster formed, and the scattering of the ensemble is consistent with the highly fluctuated RMSD values seen in Figure S3. This indicates that there is a greater instability in the GdnHCl-induced conformation relative to the other two NMR structures.

We were interested to know what led to the isolated cluster, formed in the urea-induced ensemble, which prompted us to examine further the fluctuations within specific regions of the three NMR structures. The RMSF-per-residue analysis (Figure 2d) shows that there are two specific regions in the urea-induced structure that fluctuated greatly and more so than either the solution or the GdnHCl-induced structures. These two regions correspond to β3-strand (G61~A64) of motif 2 and a loop (C109~F118) encompassing an α-helix in motif 3 as seen mapped onto the tertiary structure of the protein in Figure 2e. Incidentally, the C-td loop region also encompasses part of the de novo β-strand forming region (F116~N119) discovered in our previous work on examining HGDC aggregation in low pH condition [17]. It is interesting to note that this loop region has the potential to form β-strand in both the HGDC structure simulated under acidic condition and the urea-induced structure under physiological solution condition, as shown in Figure S4b (Supplementary Materials). This propensity for β-strand formation in the loop region is not as evident in the GdnHCl-induced structure (Figure S4c, Supplementary Materials) and is not observed in the solution structure (Figure S4a, Supplementary Materials). As for the motif 2 β3-strand (G61~A64), it is evident from Figure S4 that (in comparison to the other two NMR structures), the urea-induced structure has lost a large percentage of β-structure in this region during the simulations. The percentage of secondary structure in the motif 2 β3-strand has dropped from 85~95% range of β-structure seen in the solution and GdnHCl-induced structures down to 27~33% in the urea-induced structure.

**Figure 3:**
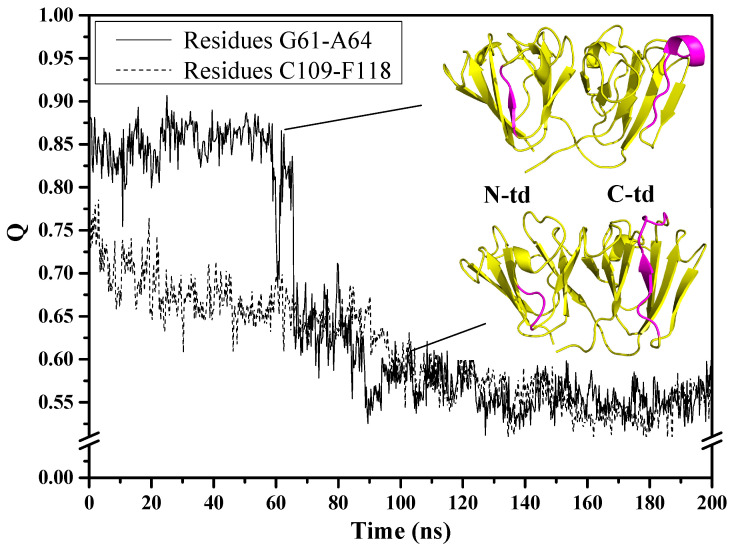
Contact fraction (Q) for specific residue regions in the urea-induced HGDC structure as a function of time in physiological solution, 343 K.

In order to understand what happened during the simulations for the two above-mentioned regions in the urea-induced structure, we monitored residue contacts of these regions with their respective neighboring residues. It can be seen from Figure 3 that the motif 2 β3-strand (G61~A64) contact fraction remained steady at 0.85 but declined quickly around 60 ns, showing a large decrease that was associated with the loss of β-structure and hydrogen bonds. The function of the hydrogen bonds was to keep this region attached to the neighboring anti-parallel β3-strand (C33~V38) of motif 1. Without these hydrogen bonds to keep it intact, the G61~A64 residue region detached from the intercalated anti-parallel β-sheet, as can be seen in Figure 3 with the lower right snapshot of the protein taken at 100 ns of simulation time. As for the motif 3 loop (C109~F118), the contact fraction at the beginning of the simulation was lower (~0.75) and steadily decreased until it reached ~0.55 around 140 ns and continued to remain at about this level till the end of simulation time. The steady loss in contact fraction indicates that the long stretch of residues did not maintain its original contacts with the surrounding residues from the beginning of the simulations. In addition, this region has the propensity to form β-strand possibly due to the conformational flexibility of the region being a loop structure and its inherent dynamic nature to adopt variable conformations.

As previously mentioned, similar to the solution structure, the GdnHCl-induced structure was able to maintain a high percentage of β-structure in motif 2 β3-strand region. However, the β-strands in other parts of the protein have shortened and became wispier in comparison to the ones in the solution structure (Figure S4a,c, Supplementary Materials). Based on these results, we can see that the denaturant has affected the integrity of some of the secondary structures, but the effects on the overall initial unfolding process are still not clear, at this point, for this particular high-energy state conformer.

### 2.3. The Overall Conformational Changes of the Denaturant-Induced HGDC Structures Simulated in 425 K

Previous studies have performed MD simulations in high temperature of 425 K and 8 M urea to study the stability and aggregation of HGDC [14,15,20]. High-temperature MD simulations have been known to accelerate protein unfolding without affecting the course of the pathway [21,22]. We, therefore, increase the temperature to 425 K to speed up the process, allowing for a further detailed look at the initial steps leading to the unfolding/misfolding/refolding pathways of the two higher energy state protein structures previously induced in the NMR experiments. Hereafter, results and discussions are only aimed at these two structures, unless otherwise specified.

To get an overall picture of the protein region(s) affected the most along the initial stages of unfolding, we re-examined the RMSF per residue of the two denaturant-induced structures and found that the range of residue fluctuation has increase from nearly 6 Å, in the urea-induced structure under 343 K (Figure 2d), to close to 7.5 Å in the GdnHCl-induced structure under 425K (Figure S5, Supplementary Materials). Although the ranges of fluctuation in both proteins have increased in the higher temperature condition, the GdnHCl-induced structure yielded overall higher fluctuation than the urea-induced structure, suggesting that the GdnHCl-induced structure has become more destabilized. This instability is more prominent in the N-terminal domain (N-td) than the C-terminal domain (C-td) where the overall fluctuation is observed to be the highest (Figure S5, Supplementary Materials).

**Figure 4:**
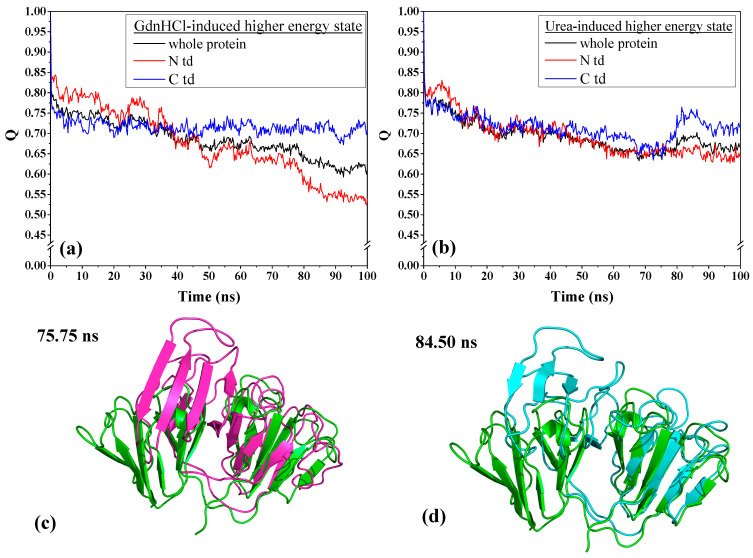
Contact fraction (Q) of HGDC whole protein and individual domains as a function time in physiological solution, 425 K, for (**a**) GdnHCl-induced and (**b**) urea-induced structures. Conformations of the urea-induced structure at (**c**) 75.75 ns (magenta) and (**d**) 84.50 ns (cyan) were superimposed by C-td to the initial conformation (green) at time zero of the MD simulations.

To understand the extent of the changes each domain has undergone, we examined the contact fraction for the whole protein and by protein domains, as depicted in Figure 4. We see that the contact fraction for the GdnHCl-induced whole protein steadily decreased until reaching close to 0.60 (Figure 4a). The contribution to this decrease by individual domains is mainly due to N-td’s loss of residue contacts. Comparing with C-td, N-td suffered the greatest loss, with contact fraction dropping down below 0.55, while it remained slightly above 0.70 in the C-td. The steepest drop in N-td residue contact occurred slightly before 80 ns, signifying that there was a drastic change in protein conformation around this time. As for the urea-induced structure, we also see a decrease in residue contacts for the whole protein and the individual domains with similar level of loss in residue contacts for the greater part of the simulations until reaching a contact fraction value of ~0.65 (Figure 4b). However, around 75 ns of simulation time, we began to see a rise in contact fraction in the C-td, which suggests that some of the lost contacts were somehow regained and the contact fraction reached a peak of ~0.75 for the C-td after 80 ns and began to level off slightly above 0.70 till the end of simulation time.

To confirm whether the urea-induced structure really did revert back toward the original higher energy state conformation, we individually superimposed the structures at 75.75 ns and 84.50 ns, corresponding to the time of the C-td contact fraction minimum and maximum, respectively, with the initial structure at time zero of the simulation. The RMSD values for the overall protein alignment yielded 8.46 Å and 7.53 Å for the structure alignments of the conformers at 75.75 ns and 84.50 ns, respectively, showing that there is a slight decrease of RMSD at the peak time. We then superimposed the structures by domain and found that despite there was not much of a difference when superimposed by N-tds (4.83 Å and 4.85 Å for structures at 75.75 ns and 84.50 ns, respectively), superposition of C-tds at the specific time frames mentioned above (Figure 4c,d) yielded 4.64 Å and 1.73 Å, respectively, signifying that at 84.50 ns, the C-td reverted back to a conformation closer to the original high-energy state structure. This is an additional support for the regained contact fraction observed in Figure 4b and shows that the urea-induced structure, when transferred into a physiological solution condition, did revert back to its original higher energy state, which may possibly be a more steady-state and more preferred conformation than the GdnHCl-induced structure.

**Figure 5:**
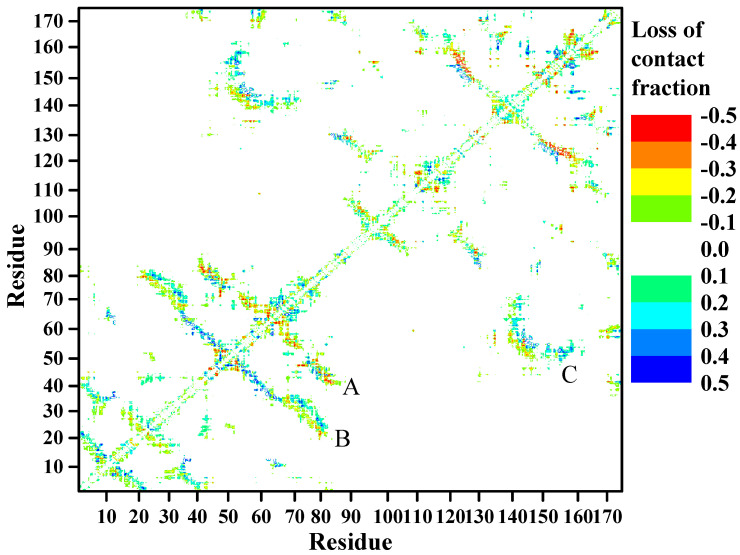
Subtractive 2D residue contact map of the denaturant-induced HGDC structures in physiological solution, 425 K, over 100 ns of simulation time. Negative loss of contact (redder hue) denotes greater loss in the urea-induced structure, positive loss (bluer hue) denotes greater loss in the GdnHCl-induced structure. Region A represents contacts between residue regions D65~M70 (motif 2 loop between β3- and β4-strands) and Q55~A64 (motif 2 β2- and β3-strands), G71~H84 (motif 2 β4-strand) and S40~N50 (motif 2 β1-strand). Region B represents contacts between regions P49~P83 (motif 2 β2-, β3-, and β4-strands) and H23~N50 (motif 1 β3-strand and motif 2 β1-strand). Region C represents contacts between N138~A162 (motif 4 β2- and β3 strands) and N50~L72 (motif 2 β2- and β3-strands).

A subtractive 2D-contact map analysis (Figure 5) was performed by subtracting the 2D residue contact map of GdnHCl-induced conformational ensembles (Figure S6b, Supplementary Materials) from that of the urea-induced ensembles (Figure S6a, Supplementary Materials) to reveal the relative changes between these two denaturant-induced higher state conformers during the simulations. Three distinctive regions of residue contact loss in the denaturant-induced structures can be seen in Figure 5. The urea-induced structure lost residue contacts mainly in motif 2, indicated by region A, while contact loss was more extensive in the GdnHCl-induced structure, which included not only motif 2, but also parts of motifs 1 and 4 depicted in regions B and C. Thus, a great proportion of the N-td in the GdnHCl-induced structure suffered contact loss (region B), as well as residue contacts in the hydrophobic inner core region (region C) that encompasses the inter-domain interface between motifs 2 and 4.

### 2.4. Examining the Critical Interactions for Maintaining Structural Stability of HGDC

The interdomain interface has been previously shown in protein refolding experiments to be important in stabilizing the final N-td conformation after the domain has folded up [23]. Based on several studies, these interface interactions have also been indicated as contributing to the high kinetic barriers during the early stages of HGDC unfolding [24,25]. Having known that the domain interface plays an important role in maintaining HGDC stability, we next examined the residue interactions in this region. Our results in Figure S7 show that the fluctuation in the interface interactions for urea-induced structure ranges from 0.2~0.8, while, for the GdnHCl-induced structure, it extends to an even wider range of 0.1~0.9, indicating that GdnHCl’s destabilizing effect is greater in this region than that of urea’s. This large fluctuation in the inter-domain contacts of the GdnHCl-induced structure indicates that the integrity of the interface has been lost; thus, breaching the high kinetic barriers and leading to structural unfolding manifested in a greater change in conformation and loss of contacts, predominantly in the N-td (see previous section). Even though the interface contacts in the urea-induced structure also fluctuate quite a bit, its effect is not as detrimental as in the GdnHCl-induced structure and is only limited to motif 2 as seen in Figure 5. Nevertheless, motif 2 is the first region affected in both denaturant-induced higher-energy NMR structures when the domain interface is disturbed. A previous MD simulations study of tyrosine-to-alanine substitution in X-ray HGDC structure also reached a similar conclusion, where it was stated that “the stability of motif 2 is mainly determined by the inter-domain interface” [20].

The fact that motif 2 is the first region to be disrupted in both denaturant-induced structure prompted us to look further into how this disruption came about. According to past studies on HGDC, it was suggested that aromatic residues are the key to maintaining the lens crystallin fold and stability [23], and some of their interactions may serve as nucleation sites for protein folding, forming “clasps” to stabilize local conformation [26]. Aromatic residues can form pairs and clusters in β-hairpin peptides and proteins (such as HGDC) containing β-hairpins to stabilize the structural fold [23,27,28]. Motif 2 in HGDC contains important aromatic residue interactions that contribute to the stability of this particular Greek key: a Tyr-pair (Y46-Y51) conserved across the βγ-crystallin superfamily and a Tyr-corner (Y63) that forms an aromatic cluster with W69 and Y56. The Greek key Tyr-pair was known to nucleate motif 2 refolding [23], while W69 (located on a surface loop region) was found to shield an aggregation-prone stretch of residues (L54~L58), predicted by bioinformatics methods [29]. Residue Y56 within this region is also a part of the N-td inter-domain interface contacts. The importance of aromatic clusters in stabilizing the Greek key fold has previously been explored. It was found that photo-oxidative damage to these residues by UV rays lead to the loss of aromatic interactions, which may contribute to cataract formation as the residues are known UV absorbers [30].

**Figure 6:**
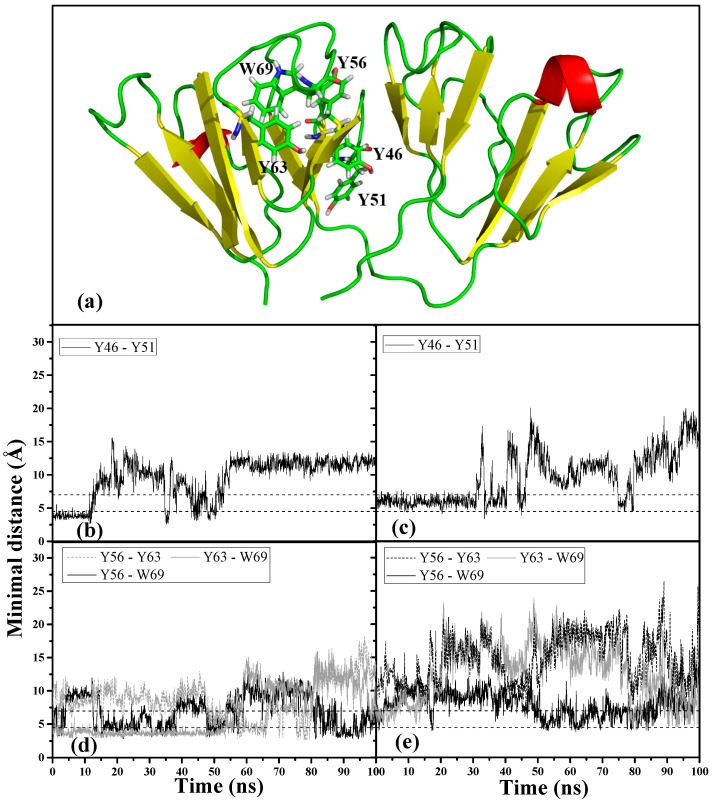
Important aromatic residues and associated interactions within motif 2. (**a**) Positions of the residues (represented by stick form) forming aromatic cluster (Y63–W69–Y56) and Tyr-pair (Y46–Y51). Minimal distance between the Tyr-pair in (**b**) urea-induced and (**c**) GdnHCl-induced structures as a function of simulation time. Minimal distance between aromatic cluster residues in (**d**) urea-induced and (**e**) GdnHCl-induced structures as a function of simulation time. Dash lines denote aromatic–aromatic residue interaction range found in most proteins [34].

We examined the interactions of the important aromatic residues within motif 2 to understand the roles they play in the dynamic conformational changes that occurred in the two denaturant-induced structures. Figure 6a shows the location of the motif 2 aromatic cluster (Y63-W69-Y56) and Greek key Y pair (Y46-Y51) within the 3D structure of HGDC. It has been observed that aromatic residues in proteins often form clusters and majority of the aromatic-aromatic interactions fall within the range of 4.5~7.0 Å [26]. Based on the above criteria, we monitored these residue interactions in motif 2. Of all the aromatic interactions observed within this Greek key motif, the conserved Tyr-pair (Y46-Y51) in both denaturant-induced higher energy structures became unstable and lost its interaction much earlier on during the simulations. As can be seen in Figure 6b,c, the Tyr-pair in the GdnHCl-induced structure started losing contact around 30 ns into the simulation, whereas the interaction in the urea structure lost contact even earlier on (~10 ns). The destabilizing effects of both denaturants were also observed to have extended to the aromatic cluster, Y63–W69–Y56, where most of the aromatic interactions were disrupted (Figure 6d,e). It is interesting to note that, of the aromatic cluster interactions, Y56–W69 maintained the highest percentage of aromatic contacts throughout the simulation time in both denaturant-induced structures (Table 1), despite its fluctuating quite a bit. This signifies that the interaction between Y56 and W69 is stronger than the rest of the interactions within the cluster, and more resistant to the chemical denaturation effects regardless of the denaturant type. W69 was found to shield the aggregation-prone N-td interface that encompasses Y56 [29]. The N-td interface forms part of the interdomain interface, a crucial region that has been shown time and time again to have significant function in keeping the two domains together and preserving the overall protein stability [31,32,33]. Based on the above, it is not difficult to understand (from the standpoint of evolutionary significance of HGDC’s function as a long-lived structural protein) that the interaction between these two residues would need to be the strongest in order to maintain structural stability and resist aggregation. Nevertheless, both denaturants disrupted the above-mentioned important aromatic interactions to the point that most of the interactions were lost toward the end of the simulation time. Despite the GdnHCl-induced structure having a slightly greater percentage of contacts than that of the urea structure for most of these aromatic interactions (as depicted in Table 1), its inter-domain interface was less stable (suffering larger fluctuation and greater loss of interface contacts) as can be seen in Figure S7.

**Figure 7:**
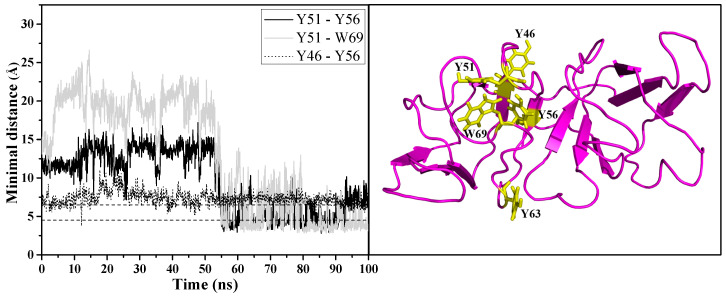
Minimal distance between the residues participating in the newly formed aromatic cluster in motif 2 of the urea-induced structure as a function of simulation time (left panel). Dash lines denote aromatic-aromatic residue interaction range found in most proteins [34]. Snapshot of the urea-induced structure taken at an 81-ns time frame (right panel) showing the relative positions of the residues (Y46, Y51, Y56, W69 in yellow, stick representation) forming the new aromatic cluster.

**Figure 8:**
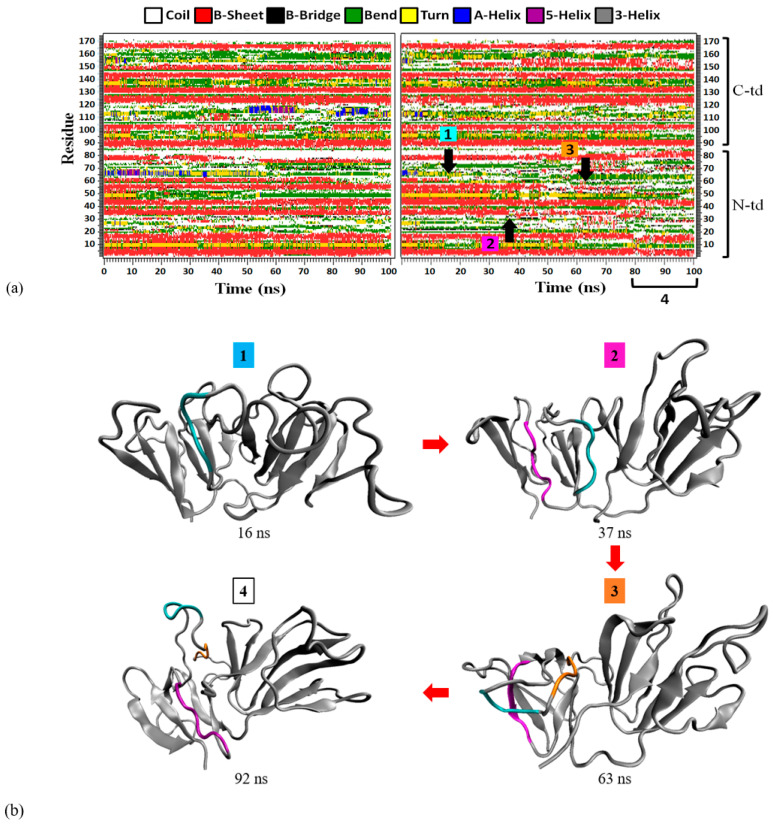
(**a**) Changes in secondary structure as a function of simulation time in the urea-induced (left panel) and GdnHCl-induced (right panel) conformers. The loss of important β-strands in the initial stages of GdnHCl-induced structural unfolding are indicated by black arrows and numbered with respect to the sequence of time with which they occurred. Number 4 indicates a range of time corresponding to near-complete melting of motif 2. (**b**) The important β-strand loss in the 3D GdnHCl-induced conformer extracted from the indicated time frame are color-coded in accordance with the color code presented in (**a**). The important β-strand loss corresponds to regions of G61~A64 (cyan), C33~V38 (magenta), and Q55~L58 (orange). A representative 3D structure indicated by number 4 shows the near-complete melting of Greek key motif 2 in the range of time corresponding to the last 20 ns of simulations.

In contrast to the GdnHCl-induced structure, the urea-induced structure was found to have formed a new non-native aromatic cluster in motif 2, as seen in the right panel of Figure 7 comprising of the following residues: Y46, Y51, Y56, and W69. Figure 7 left panel shows that the formation of the new cluster was observed around 55 ns of simulation time and these newly formed aromatic interactions remained intact until the end of the simulation time, stabilizing motif 2 and the rest of the protein structure in a semi-intact state. Not surprisingly, the greatest percentage of contacts was maintained between the interactions involving the interface residue Y56 (Y46–Y56 and Y51–Y56) as shown in Table 2; this is additional evidence supporting the importance of the interdomain interface in preserving the stability of N-td—in particular, motif 2. The more stable state of the urea-induced structure can be observed in Figure 8a (left panel) where, despite large fluctuations, most of the β-strands in the urea-induced structure (with the exception of motif 2 β3-strand, spanning residues G61~A64) were retained throughout the simulation time in comparison with the GdnHCl-induced secondary structures seen in Figure 8a (right panel). It is noteworthy to mention that the loss of β-strand in the G61~A64 residue stretch of the urea-induced structure occurred under both temperatures (343 K and 425 K) simulated in this study. This is supporting evidence indicating that the pathway that the conformer took was not altered by the higher-temperature simulation. Contrary to the urea-induced structure, we did not observe any new aromatic cluster forming in motif 2 of the GdnHCl structure. With the original aromatic interactions disrupted and without any new cluster forming to stabilize the second Greek key, motif 2 and some of the β-strands in the neighboring motifs next to it became disordered and began to lose structural integrity in the early stages of GdnHCl-induced structural unfolding.

### 2.5. Consequences of Losing Aromatic Interactions after Overcoming the High Kinetic Barriers Posed by the Interdomain Interactions in GdnHCl-Induced Structure

**Figure 9:**
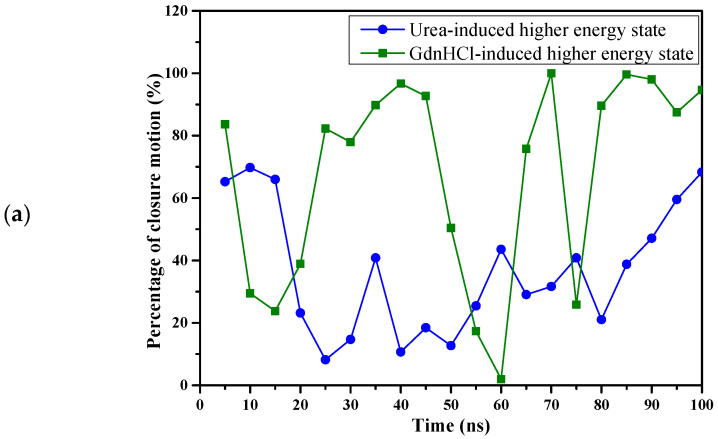

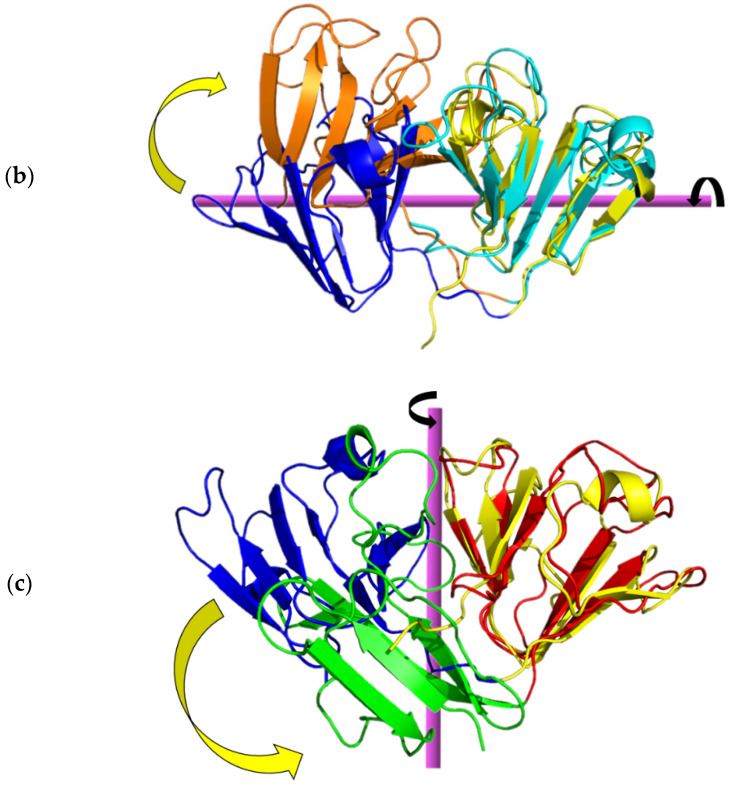
(**a**) Interdomain motions of the HGDC higher energy state structures as a function of simulation time. Superimposition of the C-td domains in (**b**) urea-induced structures and (**c**) GdnHCl-induced structures at the beginning (0 ns) and toward the end (92 ns) of the simulation time. Both the urea- and GdnHCl-induced conformers at 0 ns are colored blue (N-td) and yellow (C-td). The urea- and GdnHCl-induced structures at 92 ns are colored orange (N-td)/cyan (C-td), and green (N-td)/red (C-td), respectively. The hinge axis is presented in violet, the black arrow shows the rotational motion around the axis, and the yellow arrow indicates the motion of N-td with C-td’s of the structures at 0 ns and 92 ns superimposed.

As mentioned in the previous section, the GdnHCl-induced structure has lost more β-strands than the urea-induced structure (Figure 8a). On close observation of the right panel in Figure 9a, this destabilization of the secondary structure was mainly observed in the N-td, starting with motif 2 β3-strand (G61~A64) around 15 ns, followed by motif 1 β3-strand (C33~V38) after 35 ns, then motif 2 β2-strand (Q55~L58) after 50 ns, finally leading to the near-complete unraveling of Greek key motif 2 after 80 ns. The sequence of the structural loss is depicted in Figure 8b. Just like the urea-induced structure, we saw that the first region affected in motif 2 was the β3-strand. Therefore, we conclude that the second Greek key β3-strand region is the most vulnerable part of motif 2, prone to loss of β-structure after the protein has been brought to a higher energy state, irrespective of the denaturant type. Motif 1 β3-strand was the second β-strand to come undone, as this region forms an anti-parallel β-structure with motif 2 β3-strand in the native HGDC. Hence, it became unstable after losing its anti-parallel β3-strand partner in motif 2, even though the first Greek key was still able to maintain most of its secondary and tertiary structures during the entire period of the simulations (see Figure 8a right panel and Figure 8b). Incidentally, the motif 2 β2-strand (Q55~L58) region encompasses some of the N-td interface residues (Q55 and F57) important for keeping the two domains intact. F57 has been found to be 80% conserved across the γ-crystallins and is crucial in maintaining protein stability in both bovine GDC and HGDC based on Ala substitution site-mutagenesis experiments [5,35]. Thus, losing the secondary β-structure encompassing this residue may be the key to overcoming the high kinetic barrier of the interdomain interface in the GdnHCl-induced structure and causing it to spiral down toward the subsequent unfolding and possibly misfolding pathway.

Previous studies have proposed several hypotheses along the same line about the aggregation of partially unfolded HGDC, starting from domain swapped dimers as an initial process for cataract formation. One MD simulation study proposed that three β-strands from motif 4 of the C-td interact with the N-td of another monomer [15], while another based on single-molecule force spectroscopy suggested that β1 and β2 strands from the extruded β-hairpin loop in N-td motif 1 swap with adjacent monomers [36]. A third study, combining simulations and experiments to examine mutant HGDCs, proposed that the β1-strand of motif 1 can form anti-parallel hydrogen bonds with the β2-strand (part of the inter-domain interface) from motif 4 of C-td to form domain-swapped dimers [37].

Despite the fact that we were unable to see past the unfolding of motif 2 in either of the denaturant-induced structures examined in our study, we were able to deduce from our results that the destabilization and unfolding of the second Greek key led to the destabilization of the first Greek key. We have already witnessed this destabilizing process with motif 1 β3-strand losing secondary structure after the loss of adjacent motif 2 β3-strand (see Figure 8). Hence, our results suggest that the destabilization of motif 1 begins with the loss of β3-strand that may eventually translate into the extension of the motif 1 β-hairpin loop causing β1- and/or β2-strand to swap with an adjacent monomer [36] in the high-protein-concentration setting of the human lens. During the process, anti-parallel hydrogen bonds are formed between the extended region(s) of motif 1 and motif 4 β2-strand (part of the inter-domain interface) of the C-td [37]. This exchange of β-strands leads to the formation of domain-swapped dimers, as proposed by Garcia-Manyes et al. (2016) [36] and Serebryany et al. (2016) [37].

### 2.6. Effects of the Structural Disruptions on the Level of Interdomain Motion in HGDC

We monitored the relative motion of the two domains in the denaturant-induced structures by performing the DynDom analysis [38] and found that the denaturants exerted different rotational motion effects on the HGDC higher energy state conformers. Urea’s denaturing effect tends to cause the structure to rotate in a twist motion (63.25% of simulation time) around the horizontal axis as portrayed in Figure 9a,b with the N-td rotating into the page when C-td’s of the conformations at the beginning and close to the end of the simulation time were superimposed. On the other hand, Figure 9a shows that GdnHCl induced a closure motion (67.79% of simulation time) around a vertical axis between the domains where N-td rotates out of the page when C-td’s of the conformations at the beginning and close to the end of the simulations were superimposed, as depicted in Figure 9c. What roles these rotational motions may play further down the unfolding pathways is beyond the scope of this study. The extent of what we can see so far only fills the gap between the initiation of unfolding to what happens before N-td completely unfolds. How these rotational motions affect the process further along the pathway(s) that prompted the intermolecular association of multiple HGDCs to form aggregates, leading to the disease manifestation of cataract, warrants further investigation.

### 2.7. Relative Positions of the Simulated HGDCs in the Energy Model of the Initial Stages of Unfolding

There has been no direct evidence in the past literature on the sequence of how the HGDC N-td unfolds prior to its complete unfolding. Our study fills in this gap by exploring the much earlier unfolding process of the N-td. We were able to do so because our starting structures for the MD simulations originated from the more dynamic conformers that have undergone structural disturbance induced by denaturants in the solution NMR experiments. These disturbed conformational states (on the brink of unfolding) provided us starting points in the simulations that revealed in greater detail of the initial unfolding mechanism, allowing us to tease out the very beginning stages of HGDC unfolding. Our results showed that motif 2 in the N-td was disturbed first in the very early stages of unfolding, which was previously speculated through MD simulations of the X-ray HGDC structure (PDB: 1HK0) with Ala substitution of selective residues. Our study provided additional evidence that supported the theory of the domain interface being the key element in stabilizing the HGDC structure and showed that if the interface was disturbed enough to break the kinetic energy barrier that kept it intact (as in the GdnHCl-induced conformer), the N-td would begin to destabilize starting with the second Greek key. This finding is consistent with that of the previous Ala mutagenesis simulation study, which has proposed that the integrity of the interdomain interface is the main determinant of motif 2 stability [20]. On the other hand, the interface of the urea-induced structure was not disturbed enough to cross the high-kinetic-energy barrier necessary for unfolding to occur; therefore, it remained in a semi-intact state.

**Figure 10:**
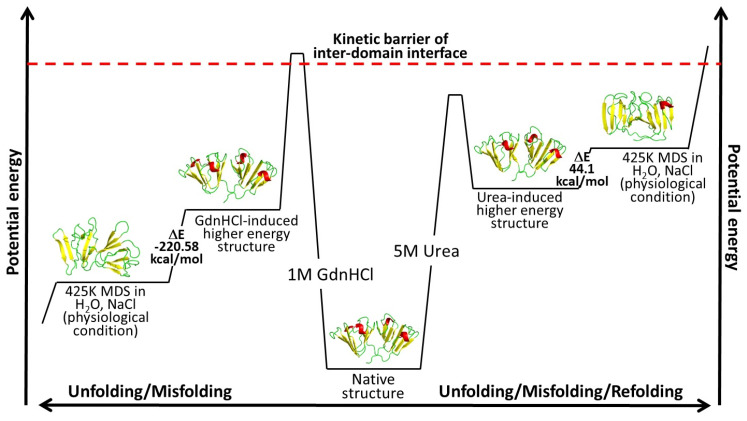
Schematic diagram of the energy model for the HGDC simulated structures with respect to the potential pathways they may undergo.

We calculated the changes in potential energy between the proteins at the beginning and the end of simulation time to understand the relative changes in potential energy experienced by the two denaturant-induced higher energy state structures simulated under 425K in a physiological solution devoid of denaturants. Based on our potential energy calculation and the observation of changes in molecular details revealed by MD simulations, we were able to deduce a more thorough potential energy model for the two higher-energy-state structures, as depicted in Figure 10. The change in potential energy from the beginning to the end of simulations was slightly positive (44.1 kcal/mol) for the urea-induced structure, while that of the GdnHCl-induced structure was a much larger negative value (−220.58 kcal/mol). This signifies that the urea-induced structure had not traversed far from its initial conformation at the beginning of the MD simulations, which was exemplified by the predominantly intact secondary structure (Figure 8a) and the two fairly well-preserved domains in their near-native conformations were maintained up till the end of the simulation time. The fact that the contact fraction at the domain interface experienced less fluctuation (Figure S7) and the overall residue contact loss was less extensive than in the GdnHCl-induced structure (Figure 5) suggested that the interface stability was still somewhat retained in the urea structure; therefore, the protein did not proceed toward the unfolding pathway. Another way to see it is that, although 5M urea may have brought the protein up to a higher-energy state, it was still not enough to break the kinetic barrier leading to the melt-down of the domain interface (Figure 10). Hence, the urea-induced higher-energy structure fell into a local minimum well and could not get out. Rolling around within the potential well, it slightly reverted back toward the original higher-energy conformation near the end of the simulation time (Figure 4a,d), thereby yielding a small positive potential energy change seen in Figure 10.

In contrast, the interdomain interface of the GdnHCl-induced structure was disturbed enough that it overcame the high-kinetic-energy barrier, leading to the destabilization of this important region; thus, causing the protein to quickly shift away from the unfavorable high-energy state to find another transitioning point, in the process leading to a great disruption in motif 2. A previous study found that the integrity of the domain interface is critical for maintaining the kinetic stability of the N-td core under physiological solution condition [33]. The GdnHCl-induced structure has evidently lost the integrity of the inter-domain interface; thereby, resulting in a great disorder in N-td, starting with motif 2. The change in overall conformation of the GdnHCl-induced higher energy structure during the MD simulations was accompanied by a negative change in potential energy, with the protein transitioning into a lower-energy state as seen in Figure 10.

At first glance, it may seem counterintuitive that the more-disturbed GdnHCl-induced structure yielded a greater negative change in energy value (−220.58 kcal/mol) than the less-disrupted urea-induced structure (44.1 kcal/mol), as it is traditionally believed that the lower (the more negative) the energy value, the more stable the conformational state [39]. However, if we extend the general concept of the free energy landscape and correlate it with our potential energy results we can explain this contradiction from the standpoint of the structural disturbance being relative to the initial GdnHCl-induced higher-energy state and that our sampling of the simulated structure at 100 ns may have captured a transitioning point from the disordered to the ultimately more ordered state of a misfolded conformation further along the pathway that serves as a fundamental building block of HGDC aggregation. Thus, the decrease in energy value signifies that the transitioning structure is moving downhill toward another basin in the energy landscape that is separated energetically from the original, natively folded state.

Despite the fact that the denaturant-induced structures were artificially synthesized from in vitro experiments and not the naturally occurring states present in the human lens, one cannot rule out that in the natural lens, disruptive environmental factors may possibly produce unstable HGDC conformers not unlike the denaturant-induced ones seen in our study. The 1M GdnHCl concentration used in this study was not enough to denature the protein, but only to bring it up to a slightly higher energy state (Figure 1e), possibly to the brink of unfolding. Previous refolding experiments have found that HGDC can be refolded into a native-like state in physiological conditions without the presence of chaperones when diluted from 5 M to 1M GdnHCl. However, once the concentration drops below 1 M, the partially unfolded HGDCs began to form high-molecular-weight aggregates [40]. In our study, we extracted the 1 M GdnHCl-induced higher-energy-state NMR structure of HGDC and submitted it to MD simulations in physiological solution conditions (pH 7, 136.7 mM NaCl). In contrast to the much milder dilution refolding experiments, the drastic environmental change (from 1M GdnHCl to physiological solution conditions) created in our study pushed the higher-energy conformer even further away from the native-fold. Based on our results, we believe that if the initial unfolding conformer (similar to the GdnHCl-induced structure observed in our study) does exist at some point in the lifetime of a HGDC within the high protein concentration environment of the human lens, we would expect to see it to either continue down the unfolding or misfolding pathway. Along the path, the non-native structure has the potential to form intermolecular association with neighboring crystallin proteins, and ultimately aggregating within the lens leading to cataract formation.

## 3. Materials and Methods

### 3.1. Materials

Salts were obtained from Fluka Honeywell. Tryptone and yeast extract were purchased from Conda (Torrejón de Ardoz, Madrid, Spain). Chromatography columns were obtained from Sigma-Aldrich (St. Louis, Missouri, USA). Kanamycin, imidazole, and isopropyl β-D-thiolgalactorpyranoside (IPTG) were purchased from Biobasic (Markham, Canada). Toyopearl AF-Chelate-650M resin was purchased from Tosoh (Tokyo, Japan). All other chemicals were of reagent grade and obtained from Sigma-Aldrich (St. Louis, MO, USA) unless otherwise specified. ^15^N-labeld ammonium chloride and ^13^C-labeled D-glucose were purchased from Sigma-Aldrich (St. Louis, MO, USA) and Cambridge Isotope Laboratories, Inc (Tewksbury, MA, USA), respectively.

### 3.2. Cloning, Expression, and Purification of HGDC Protein

The 6×His-HGDC gene fragment from plasmid pQE1 and the digested plasmid pET-30b(+) was used for the generation of pET30b-HGDC. The recombinant protein HGDC was expressed and produced using the *E. coli* strain BL21(DE3). The detailed procedures and conditions of expression and purification of HGDC have been described previously [18,41].

### 3.3. NMR Sample Preparation and Spectroscopy

For the NMR experiments, the concentration of HGDC solution was brought to ~0.3 mM by centrifugal ultrafiltration (MWCO of 15,000 Da, Merck Millipore, Burlington, Massachusetts, USA), and D_2_O (10% *v*/*v*) was added to the sample solution. Sodium trimethylsilylpropionate (TSP) was used as an internal standard of chemical shift. In addition, two other HGDC sample solutions with 5.0 M urea and 1.0 GdnHCl were prepared for the NMR structural studies. The final sample solutions were transferred to 5-mm NMR tubes (Shigemi Co., Tokyo, Japan) for NMR measurements. To measure the residual dipolar couplings (RDCs), the ^15^N-labeled HGDC solution (in the absence or presence of denaturants) was soaked into 4% polyacrylamide gels (the ratio of acrylamide to bis-acrylamide is 29:1) in a 5.4-mm chamber (New Era Enterprises, Inc., Vineland, NJ, USA) for at least 24 h at 4 °C, then transferred to a Wilmad open-ended NMR tube [34]. NMR experiments were performed at 298 K on a Bruker AVANCE-500 spectrometer equipped with a 5-mm inverse triple resonance, Z-axis gradient probe, or on Bruker AVANCE-600 and 800 spectrometers equipped with 5-mm inverse triple-resonance, Z-axis gradient cryo-probes. Resonance assignments were accomplished using the following heteronuclear 3D spectra: HNCA, HNCO, HN(CO)CA, CBCA(CO)NH, HBHA(CO)NH. The backbone chemical shifts of H^α^, H^N^, C^α^, C^β^, C’ and N^H^ were measured from the assigned heteronuclear 3D spectra [42,43]. The ^1^D_N-HN_ residual dipolar coupling constants (RDCs) of HGDC in the presence or absence of denaturants were measured from the DSSE-HSQC spectra [44]. All NMR spectra were processed using the program TopSpin 2.1 (Bruker, Germany) and analyzed using AURELIA 3.9 (Bruker, Germany).

### 3.4. Generation of HGDC Solution Structure in the Absence and Presence of Denaturants

The solution structures of HGDC in the absence and presence of denaturants were first generated by GeNMR webserver (http://www.genmr.ca/index.php, accessed on 31 January 2018) [45] using all available backbone chemical shifts as input. The lowest energy structures of HGDC in the absence and presence of denaturants generated by the GeNMR webserver were used as the initial structures for further structural refinement. The dihedral angles of all residues in the initial structures were calculated using PyMOL v.2.4.0 (Schrödinger, Inc., New York, NY, USA). The medium-range distances among the inter-β strands (distances between all H^α^-H^α^, H^α^-H^N^, H^N^-H^N^ among inter-β strands with 3.5-Å distance cutoff) were calculated from the initial structures using MOLMOL v.1.7.0 (Zurich, Switzerland) [46]. The initial structures generated by the GeNMR webserver were refined by XPLOR-NIH v.2.36 (Bethesda, MD, USA) [47,48] using the experimentally measured RDCs (error range 1.0 Hz), the calculate dihedral angles (error range 20°) and the calculated medium-range distances (error range 1 Å) among the inter-β strands as restraints. A total of 100 structures were generated and the 20 lowest-energy structures were selected as the final structural ensemble of HGDC in the absence and presence of denaturants. The final solution structures of HGDC were validated by comparing the experimental RDCs with the RDCs back-calculated from the refined structures using Module 2 software (Grenoble, France) [49].

### 3.5. Molecular Dynamics Simulations and Trajectory Analysis

The initial structures for molecular dynamics simulations (MD simulations) were obtained from NMR spectroscopy as previously described under solution and different denaturing conditions (5 M urea and 1 M GdnHCl). MD simulations for the three NMR structures were conducted using GROMACS-4.5.3 with GROMOS96 G45a3 force field [50]. A 7.0 nm × 7.0 nm × 7.0 nm box was constructed and solvated with water molecules using the SPC water model [51]. Fifty-six water molecules were replaced with 28 sodium and 28 chloride ions to simulate the experimental condition of 136.7 mM NaCl. The system was energy-minimized with a time step of 2 fs and NPT conditions under 1 bar pressure and 343 K by using the steepest descent method with tolerance energy of 100 kJ/mol-nm. The particle–mesh Ewald (PME) method was used for the long-range electrostatic interactions with a grid spacing of 0.12 nm and an interpolation order of 4 The Lennard–Jones interactions were treated with a cut-off distance of 1.4 Å. Bond length was restricted by utilizing the LINCS algorithm, and periodic boundary condition (PBC) was applied to eliminate the boundary effect. After performing positional restraints for 1 ns, 200-ns simulations were conducted at 343 K for all systems with additional 100-ns runs performed under 425 K for the NMR structures obtained from samples with denaturants. Except for the temperature increase, all other simulation parameters were kept the same as the lower-temperature runs. One MD simulation trajectory of 200 ns at 343 K was obtained for each of the lowest energy conformers (in solution, 5 M urea, and 1M GdnHCl) extracted from NMR spectroscopy. The additional 100-ns higher-temperature trials at 425 K were replicated at least three times for the initial NMR urea- and GdnHCl-induced higher-energy structures to confirm that the general trend of structural changes were present for each of the conformers before performing further data analysis on the most representative trajectory for each of the denaturant-induced structures.

The analysis of backbone root-mean-square-deviation (RMSD), backbone root-mean-square fluctuation (RMSF), hydrogen bonds, distance between selected residue regions, and secondary structure prediction based on DSSP [52] were performed through the built-in protocols set within the Gromacs program. The MDAnalysis module was implemented for contact fraction analysis [53]. A contact between residues *i* and *j* was counted if any heavy atom of residue *i* was within 6.5 Å of any heavy atom of residue *j*. The contact fraction (Q) is a unit-less number ranging from 0 to 1 and is calculated as the total number of contacts counted in each time frame divided by the total number of contacts within the initial reference structure at time zero (t = 0) of the simulation.

Principal components analysis (PCA), a covariance matrix-based statistical technique, was used to examine the global and correlated motion in simulation trajectories [54]. By diagonalizing the covariance matrix of the atomic coordinates obtained from the MD simulations [55], the eigenvectors and eigenvalues of the systems were calculated and sorted. The simulation trajectories were mapped on to the first and second eigenvectors with the top two eigenvalues with respect to the main chain heavy atoms to filter out the random intra-molecular fluctuations and pinpoint the significant large-scale protein motions.

The intra-molecular interactions were identified using the Protein Interaction calculator (PIC) webserver (http://pic.mbu.iisc.ernet.in, accessed on 23 February 2021) [56] along the time step of 5 ns. Interdomain motion was analyzed by Dyndom (http://dyndom.cmp.uea.ac.uk/dyndom, accessed on 1 February 2021) [57,58,59]. Dyndom determines interdomain rotation, hinge axes, and percentage of closure motion between the initial structure and a structure at designated time. Inter-domain orientation was visualized by VMD-v1.9.3 visualization software (Urbana, IL, USA) [60] and PyMOL v.2.4.0 (Schrodinger Inc., New York, NY, USA).

### 3.6. Calculation of Changes in Potential Energy for the Simulated Structures of HGDC

The potential energy of simulated structures was calculated by using Dreiding energy calculation [61] implemented in Discovery Studio 2017 R2 (Biovia, San Diego, CA, USA). The Dreiding force field utilizes a general force constant and geometry parameter based on simple hybridization. In brief, the energy terms in the potential energy calculation are expressed in following Equation (1):

*E_pot_* = *E_valence_* + *E_nonbond_* (1)

where the potential energy for a protein molecule is the sum of valence bonded interactions (*E_valence_*) and non-bonded interactions (*E_nonbond_*). The valence bonded interaction is, in turn, expressed as Equation (2):

*E_valence_* = *E_str_* + *E_bend_* + *E_tor_* + *E_inv_* (2)

where *E_str_* denotes bond stretch, *E_bend_* bond-angle bend, *E_tor_* dihedral angle torsion, and *E_inv_* inversion terms. *E_inv_* accounts for the inversion energy required to restore proper planar configuration for certain bonds. The non-bonded interaction is described as follows in Equation (3):

*E_nonbond_* = *E_van_* + *E_elec_* + *E_hbond_* (3)

where *E_van_* denote van der Waal, *E_elec_* electrostatic, and *E_hbond_* hydrogen bond interactions. Further detail of the Dreiding energy calculation can be found in the work by Mayo SL and coworkers (1990) [61].

The calculation of change in potential energy (Δ*E*) for the simulated structures during the 425 K simulations is as follows in Equation (4):

Δ*E = E*_t100_ − *E*_t0_ (4)

where *E*_t100_ is the potential energy of the simulated structure at the end of the simulation time (at 100 ns) and *E*_t0_ is that of the initial structure at time zero. The structures used for the changes in energy calculations were the initial NMR structures of the urea- and GdnHCl-induced higher-energy-state conformations at time zero before proceeding with the 425-K MD simulations and their respective final structural counterparts extracted at the end of the 100 ns simulations.

## 4. Conclusions

Our observation from the MD simulations of the two denaturant-induced higher-energy NMR structures leads us to conclude that the initial destabilization of HGDC begins with the interdomain interface, consistent with what was reported in the past literature [25,32,33], followed by the disruption in motif 2 starting with the melt-down of β3-strand in the second Greek key. If the disruptive force does not break the high kinetic barrier of the interface, the conformer may be left in an intermediate semi-steady state within a potential well, as seen with our urea-induced structure. This particular conformer may take any of the three possible pathways (refolding, unfolding, or misfolding) if disruptive energy is high enough to break it out of the local minimum. On the contrary, if the disruptive force is great enough to surpass the high-kinetic-energy barrier that keeps the interface intact, the N-td would continue to unfold in the sequential order of β3-strand (first Greek key) followed by β2-strand (second Greek key), until the destabilizing effect of motif 2 spreads to motif 1. Hence, the conformer, as seen with our GdnHCl-induced structure, may continue down the unfolding or misfolding pathway and participate in forming intermolecular interactions that include domain swapping with neighboring HGDCs in the high-protein-concentration environment, as described in previous reports [36,37]. Figure 10 summarizes the above-mentioned description of our results, which enables us to obtain a general consensus (from two possible HGDC conformers) of what effects unfavorable or destabilizing environmental factors may have in thwarting the structural stability of HGDC and causing it to spiral down the aggregation pathway—the basis for the pathogenesis of cataract.

Previously, Serebryany et al. (2016) [37] have suggested that a potential cataract intervention strategy is to inhibit the aggregation of HGDC by blocking the binding edge of motif 4 β2-strand (part of the interdomain interface) with a peptide or small-molecule inhibitor to compete with the N-td β-hairpin or target a specific part of the native structure if the origin of the domain swap came from a partially misfolded state. Based on the results of our study, we expand this concept of early cataract intervention to include binding sites in motif 2. Small stabilizing compounds can be designed to stop the second Greek key β3-strand from unraveling and to maintain the native intra-motif aromatic–aromatic contacts, as these interactions are critical in keeping the Greek key motif intact. Enhancing the stability of the key elements (the interdomain interface and motif 2) involved in the very early stages of HGDC unfolding can help preserve the integrity of the entire protein and extend its lifetime as a structural protein to maintain clarity of the eye lens and serve as a preventative measure to delay the onset of age-related cataract.

## Figures and Tables

**Table 1:** Percentage of the important aromatic–aromatic interactions present within motif 2 of the denaturant-induced higher-energy-state structures throughout 100 ns simulation time, 425 K.

Aromatic Interaction	Y46–Y51	Y56–W69	Y63–W69	Y56–Y63
urea-induced higher energy state (%)	8.05	28.44	5.85	15.19
GdnHCl-induced higher energy state (%)	16.19	34.98	10.59	1.90

**Table 2:** Percentage of aromatic–aromatic interactions present in the new aromatic cluster formed in motif 2 of the urea-induced higher-energy-state structure during 100 ns of simulation time, 425 K.

Aromatic Interaction	Y51–Y56	Y51–W69	Y46–W69	Y46–Y56
Urea-induced higher energy state (%)	21.54	10.34	0.25	32.68

## Data Availability

Can be provided by the authors upon request.

## Supplementary Materials

The following are available online at https://www.mdpi.com/article/10.3390/ijms23031591/s1.

## References

[1] Horwitz J. (1992). Alpha-crystallin can function as a molecular chaperone. Proc. Natl Acad. Sci. USA.

[2] Ji F.L., Jung J., Koharudin L.M.I., Gronenborn A.M. (2013). The Human W42R gamma D-Crystallin Mutant Structure Provides a Link between Congenital and Age-related Cataracts. J. Biol. Chem..

[3] Ji F.L., Koharudin L.M.I., Jung J., Gronenborn A.M. (2013). Crystal structure of the cataract-causing P23T D-crystallin mutant. Proteins.

[4] Basak A., Bateman O., Slingsby C., Pande A., Asherie N., Ogun O., Benedek G.B., Pande J. (2003). High-resolution X-ray crystal structures of human gammaD crystallin (1.25 A) and the R58H mutant (1.15 A) associated with aculeiform cataract. J. Mol. Biol..

[5] Flaugh S.L., Kosinski-Collins M.S., King J. (2005). Interdomain side-chain interactions in human gamma D crystallin influencing folding and stability. Protein Sci..

[6] Chen J., Callis P.R., King J. (2009). Mechanism of the Very Efficient Quenching of Tryptophan Fluorescence in Human gamma D- and gamma S-Crystallins: The gamma-Crystallin Fold May Have Evolved to Protect Tryptophan Residues from Ultraviolet Photodamage. Biochemistry.

[7] Jaenicke R. (1999). Stability and folding of domain proteins. Prog. Biophys. Mol. Bio..

[8] Jahn T.R., Radford S.E. (2005). The Yin and Yang of protein folding. Febs J..

[9] Greene A.E., Miller R.C., Coriell L.L. (1975). Cell-Cultures with Chromosomal Aberrations July 1974-Available from Human-Genetic-Mutant-Cell-Repository. Cytogenet. Cell. Genet..

[10] Makhatadze G.I., Privalov P.L. (1992). Protein Interactions with Urea and Guanidinium Chloride—A Calorimetric Study. J. Mol. Biol..

[11] O’Brien E.P., Dima R.I., Brooks B., Thirumalai D. (2007). Interactions between hydrophobic and ionic solutes in aqueous guanidinium chloride and urea solutions: Lessons for protein denaturation mechanism. J. Am. Chem. Soc..

[12] Khan S.H., Prakash A., Pandey P., Lynn A.M., Islam A., Hassan M.I., Ahmad F. (2019). Protein folding: Molecular dynamics simulations and in vitro studies for probing mechanism of urea- and guanidinium chloride-induced unfolding of horse cytochrome-c. Int. J. Biol. Macromol..

[13] Canchi D.R., Garcia A.E. (2013). Cosolvent effects on protein stability. Annu. Rev. Phys. Chem..

[14] Das P., King J.A., Zhou R.H. (2010). Beta-strand interactions at the domain interface critical for the stability of human lens gamma D-crystallin. Protein Sci..

[15] Das P., King J.A., Zhou R.H. (2011). Aggregation of gamma-crystallins associated with human cataracts via domain swapping at the C-terminal beta-strands. Proc. Natl. Acad. Sci. USA.

[16] Fernandez-Silva A., French-Pacheco L., Rivillas-Acevedo L., Amero C. (2020). Aggregation pathways of human gamma D crystallin induced by metal ions revealed by time dependent methods. PeerJ.

[17] Chang C.K., Wang S.S.S., Lo C.H., Hsiao H.C., Wu J.W. (2017). Investigation of the early stages of human D-crystallin aggregation process. J. Biomol. Struct. Dyn..

[18] Wu J.W., Chen M.E., Wen W.S., Chen W.A., Li C.T., Chang C.K., Lo C.H., Liu H.S., Wang S.S. (2014). Comparative analysis of human gammaD-crystallin aggregation under physiological and low pH conditions. PLoS ONE.

[19] Corpas L., Hadaruga N.G., David I., Pirsan P., Hadaruga D.I., Isengard H.D. (2014). Karl Fischer Water Titration-Principal Component Analysis Approach on Wheat Flour. Food Anal. Method.

[20] Yang Z.X., Xia Z., Huynh T., King J.A., Zhou R.H. (2014). Dissecting the contributions of beta-hairpin tyrosine pairs to the folding and stability of long-lived human gamma-crystallins. Nanoscale.

[21] Day R., Bennion B.J., Ham S., Daggett V. (2002). Increasing temperature accelerates protein unfolding without changing the pathway of unfolding. J. Mol. Biol..

[22] Beck D.A.C., Daggett V. (2004). Methods for molecular dynamics simulations of protein folding/unfolding in solution. Methods.

[23] Kong F.R., King J. (2011). Contributions of aromatic pairs to the folding and stability of long-lived human gamma D-crystallin. Protein Sci..

[24] Serebryany E., King J.A. (2014). The beta gamma-crystallins: Native state stability and pathways to aggregation. Prog. Biophys. Mol. Bio..

[25] Sahin E., Jordan J.L., Spatara M.L., Naranjo A., Costanzo J.A., Weiss W.F., Robinson A.S., Fernandez E.J., Roberts C.J. (2011). Computational Design and Biophysical Characterization of Aggregation-Resistant Point Mutations for gamma D Crystallin Illustrate a Balance of Conformational Stability and Intrinsic Aggregation Propensity. Biochemistry.

[26] Burley S.K., Petsko G.A. (1985). Aromatic-Aromatic Interaction—A Mechanism of Protein-Structure Stabilization. Science.

[27] Yao J., Dyson H.J., Wright P.E. (1994). Three-dimensional structure of a type VI turn in a linear peptide in water solution. Evidence for stacking of aromatic rings as a major stabilizing factor. J. Mol. Biol..

[28] Wu L., McElheny D., Takekiyo T., Keiderling T.A. (2010). Geometry and Efficacy of Cross-Strand Trp/Trp, Trp/Tyr, and Tyr/Tyr Aromatic Interaction in a beta-Hairpin Peptide. Biochemistry.

[29] Hsueh S.S., Lu J.H., Wu J.W., Lin T.H., Wang S.S.S. (2021). Protection of human gamma D-crystallin protein from ultraviolet C-induced aggregation by ortho-vanillin. Spectrochim Acta A.

[30] Grossweiner L.I. (1984). Photochemistry of proteins: A review. Curr. Eye Res..

[31] Flaugh S.L., Kosinski-Collins M.S., King J. (2005). Contributions of hydrophobic domain interface interactions to the folding and stability of human gamma D-crystallin. Protein Sci..

[32] Mills I.A., Flaugh S.L., Kosinski-Collins M.S., King J.A. (2007). Folding and stability of the isolated Greek key domains of the long-lived human lens proteins gamma D-crystallin and gamma S-crystallin. Protein Sci..

[33] Mills-Henry I.A., Thol S.L., Kosinski-Collins M.S., Serebryany E., King J.A. (2019). Kinetic Stability of Long-Lived Human Lens gamma-Crystallins and Their Isolated Double Greek Key Domains. Biophys. J..

[34] Chou J.J., Gaemers S., Howder B., Louis J.M., Bax A. (2001). A simple apparatus for generating stretched polyacrylamide gels, yielding uniform alignment of proteins and detergent micelles. J. Biomol. Nmr..

[35] Palme S., Slingsby C., Jaenicke R. (1997). Mutational analysis of hydrophobic domain interactions in gamma B-crystallin from bovine eye lens. Protein Sci..

[36] Garcia-Manyes S., Giganti D., Badilla C.L., Lezamiz A., Perales-Calvo J., Beedle A.E.M., Fernandez J.M. (2016). Single-molecule Force Spectroscopy Predicts a Misfolded, Domain-swapped Conformation in human D-Crystallin Protein. J. Biol. Chem..

[37] Serebryany E., Woodard J.C., Adkar B.V., Shabab M., King J.A., Shakhnovich E.I. (2016). An Internal Disulfide Locks a Misfolded Aggregation-prone Intermediate in Cataract-linked Mutants of Human gamma D-Crystallin. J. Biol. Chem..

[38] Hayward S., Lee R.A. (2002). Improvements in the analysis of domain motions in proteins from conformational change: DynDom version 1.50. J. Mol. Graph. Model..

[39] Dee D.R., Woodside M.T. (2016). Comparing the energy landscapes for native folding and aggregation of PrP. Prion.

[40] Kosinski-Collins M.S., King J. (2003). In vitro unfolding, refolding, and polymerization of human gamma D crystallin, a protein involved in cataract formation. Protein Sci..

[41] Wen W.S., Hsieh M.C., Wang S.S.S. (2011). High-level expression and purification of human gamma D-crystallin in Escherichia coli. J. Taiwan Inst. Chem. E.

[42] He K.C., Chen Y.R., Liang C.T., Huang S.J., Tzeng C.Y., Chang C.F., Huang S.J., Huang H.B., Lin T.H. (2020). Conformational Characterization of Native and L17A/F19A-Substituted Dutch-Type beta-Amyloid Peptides. Int. J. Mol. Sci..

[43] Leopold M.F., Urbauer J.L., Wand A.J. (1994). Resonance assignment strategies for the analysis of NMR spectra of proteins. Mol. Biotechnol..

[44] Cordier F., Dingley A.J., Grzesiek S. (1999). A doublet-separated sensitivity-enhanced HSQC for the determination of scalar and dipolar one-bond J-couplings. J. Biomol. Nmr..

[45] Berjanskii M., Tang P., Liang J., Cruz J.A., Zhou J., Zhou Y., Bassett E., MacDonell C., Lu P., Lin G. (2009). GeNMR: A web server for rapid NMR-based protein structure determination. Nucleic Acids Res..

[46] Koradi R., Billeter M., Wuthrich K. (1996). MOLMOL: A program for display and analysis of macromolecular structures. J. Mol. Graphics.

[47] Schwieters C.D., Kuszewski J.J., Tjandra N., Clore G.M. (2003). The Xplor-NIH NMR molecular structure determination package. J. Magn. Reson..

[48] Schwieters C.D., Kuszewski J.J., Clore G.M. (2006). Using Xplor-NIH for NMR molecular structure determination. Prog. Nucl. Mag Res..

[49] Dosset P., Hus J.C., Blackledge M., Marion D. (2000). Efficient analysis of macromolecular rotational diffusion from heteronuclear relaxation data. J. Biomol. Nmr..

[50] Schuler L.D., Daura X., Van Gunsteren W.F. (2001). An improved GROMOS96 force field for aliphatic hydrocarbons in the condensed phase. J. Comput. Chem..

[51] Berweger C.D., Vangunsteren W.F., Mullerplathe F. (1995). Force-Field Parametrization by Weak-Coupling—Reengineering Spc Water. Chem. Phys. Lett..

[52] Kabsch W., Sander C. (1983). Dictionary of Protein Secondary Structure—Pattern-Recognition of Hydrogen-Bonded and Geometrical Features. Biopolymers.

[53] Michaud-Agrawal N., Denning E.J., Woolf T.B., Beckstein O. (2011). Software News and Updates MDAnalysis: A Toolkit for the Analysis of Molecular Dynamics Simulations. J. Comput. Chem..

[54] Maisuradze G.G., Liwo A., Scheraga H.A. (2009). Principal component analysis for protein folding dynamics. J. Mol. Biol..

[55] David C.C., Jacobs D.J. (2014). Principal Component Analysis: A Method for Determining the Essential Dynamics of Proteins. Protein Dynamics.

[56] Tina K.G., Bhadra R., Srinivasan N. (2007). PIC: Protein Interactions Calculator. Nucleic Acids Res..

[57] Veevers R., Hayward S.J.B. (2019). Methodological improvements for the analysis of domain movements in large biomolecular complexes. Physicobiology.

[58] Girdlestone C., Hayward S. (2016). The DynDom3D webserver for the analysis of domain movements in multimeric proteins. J. Comput. Biol..

[59] Poornam G.P., Matsumoto A., Ishida H., Hayward S. (2009). A method for the analysis of domain movements in large biomolecular complexes. Proteins Struct. Funct. Bioinform..

[60] Humphrey W., Dalke A., Schulten K. (1996). VMD: Visual molecular dynamics. J. Mol. Graph..

[61] Mayo S.L., Olafson B.D., Goddard W.A. (1990). Dreiding—A Generic Force-Field for Molecular Simulations. J. Phys. Chem..

