# Supplementary material for: Insights to Human γD-Crystallin Unfolding by NMR Spectroscopy and Molecular Dynamics Simulations
Source: Int J Mol Sci. 2022 Jan 29;23(3):1591. doi: 10.3390/ijms23031591 (PMC8836049; doi:10.3390/ijms23031591)
Supplement: Supplementary file 1 [file ijms-23-01591-s001.zip › ijms-1505264-supplementary.pdf]

Supplementary Information

Insights to Human γD-Crystallin Unfolding by NMR Spectroscopy and Molecular Dynamics Simulations

Shu-Shun Hsueh[1,#], Steven S.-S. Wang[1,#], Shu-Han Chen[1], Chia-Lin Wang[2], Josephine W. Wu[3,*], Ta-Hsien Lin[2,4,*]

[1] Department of Chemical Engineering, National Taiwan University, Taipei 10617, Taiwan

[2] Laboratory of Nuclear Magnetic Resonance, Medical Research Department, Taipei Veterans General Hospital, Taipei 11217, Taiwan

[3] Department of Optometry, Yuanpei Medical University of Technology, Hsinchu City 30015, Taiwan

[4] Institute of Biochemistry and Molecular Biology, National Yang Ming Chiao Tung University, Taipei 11221, Taiwan

#: Both authors contributed equally to this work.

Corresponding Author:

Ta-Hsien Lin, Ph.D.

Laboratory of Nuclear Magnetic Resonance, Medical Research Department, Taipei Veterans General Hospital Taipei 11217, Taiwan

&

Institute of Biochemistry and Molecular Biology, National Yang Ming Chiao Tung University, Taipei 11221, Taiwan

Phone: +886-2-28712121 Ext.2703

Email: thlin@vghtpe.gov.tw

Corresponding Author:

Josephine Wan-Ru Wu, O.D., Ph.D.

Department of Optometry, Yuanpei Medical University of Technology

Hsinchu City 30015, Taiwan

Phone: +886-3-538-1183 Ext. 8608

Email: jwwu106104@mail.ypu.edu.tw

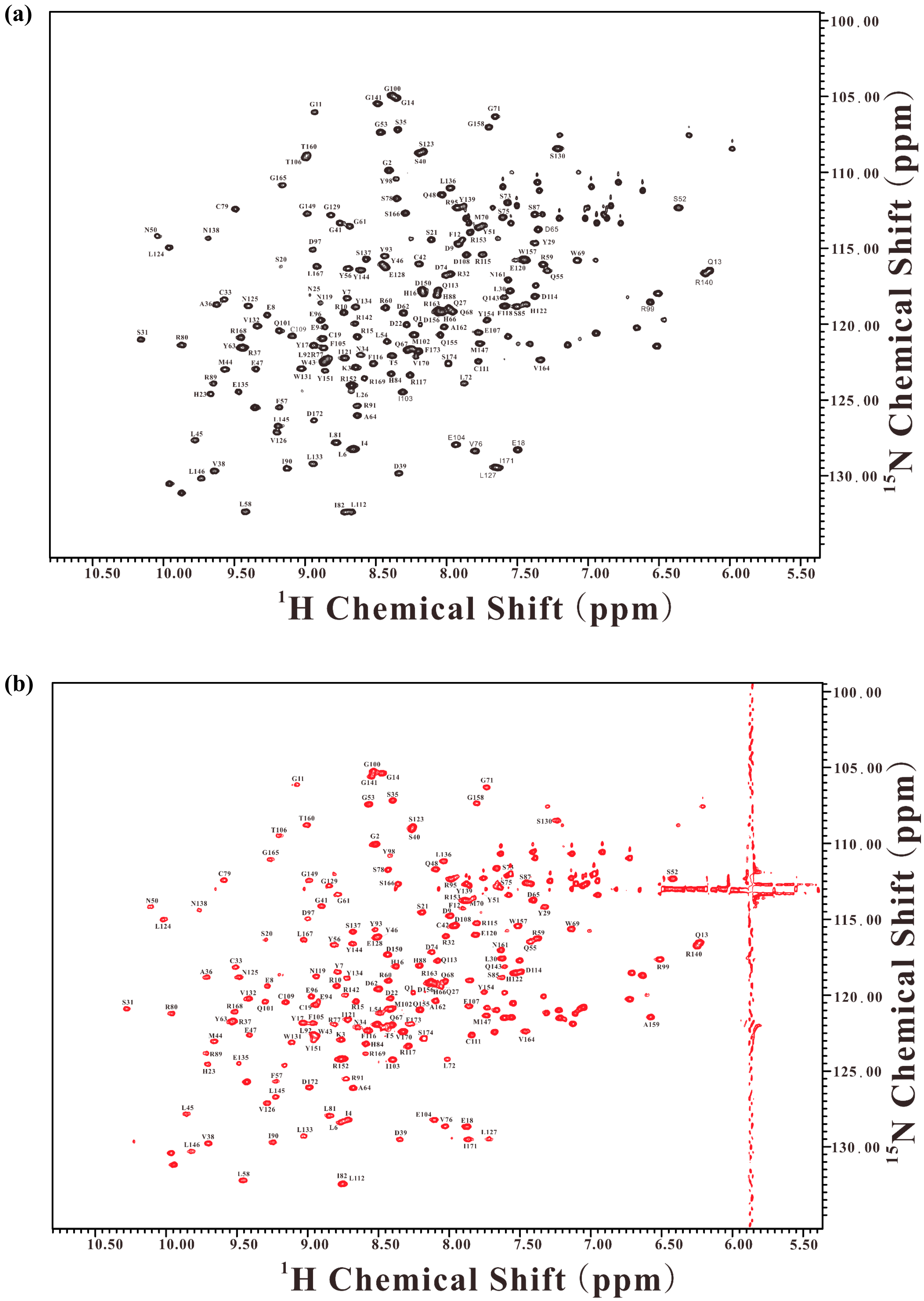
(a)
100.00
105.00
110.00
115.00
120.00
125.00
130.00
10.50 10.00 9.50 9.00 8.50 8.00 7.50 7.00 6.50 6.00 5.50
^{1}H Chemical Shift (ppm)
^{15}N Chemical Shift (ppm)
(b)
100.00
105.00
110.00
115.00
120.00
125.00
130.00
10.50 10.00 9.50 9.00 8.50 8.00 7.50 7.00 6.50 6.00 5.50
^{1}H Chemical Shift (ppm)
^{15}N Chemical Shift (ppm)

(c)

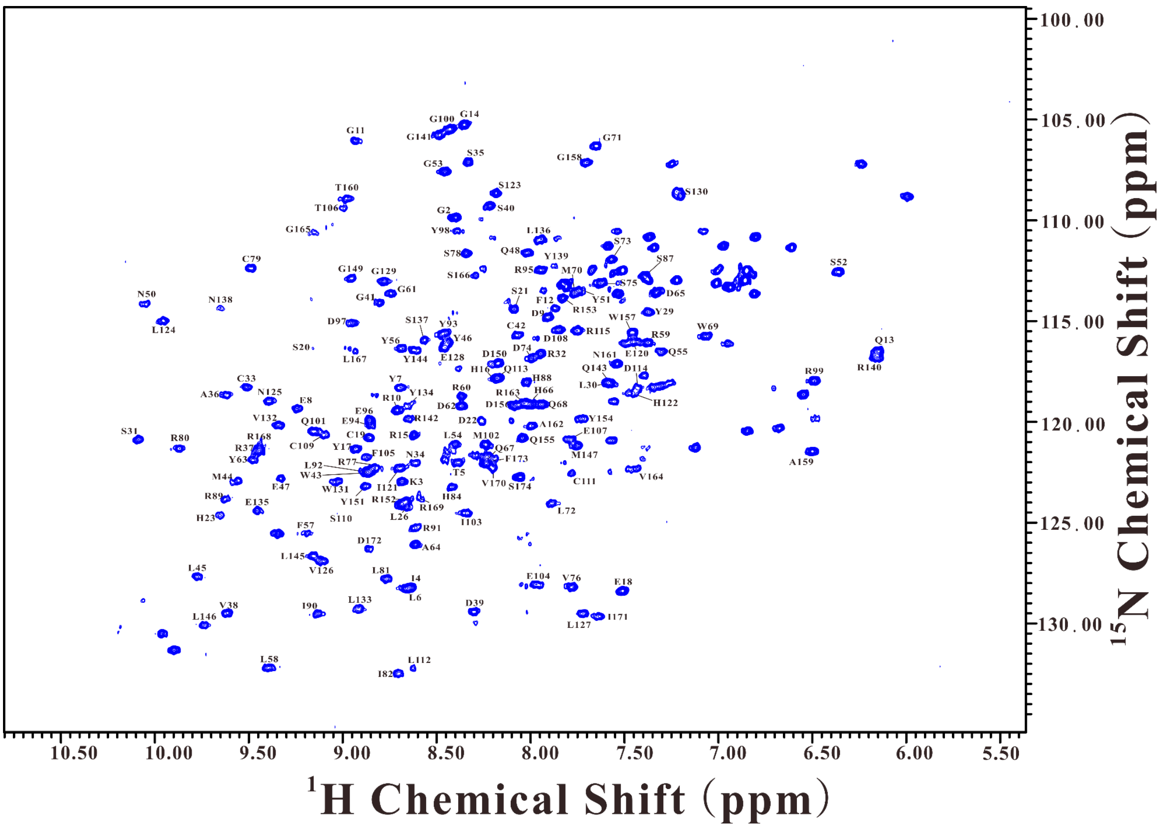

(d)

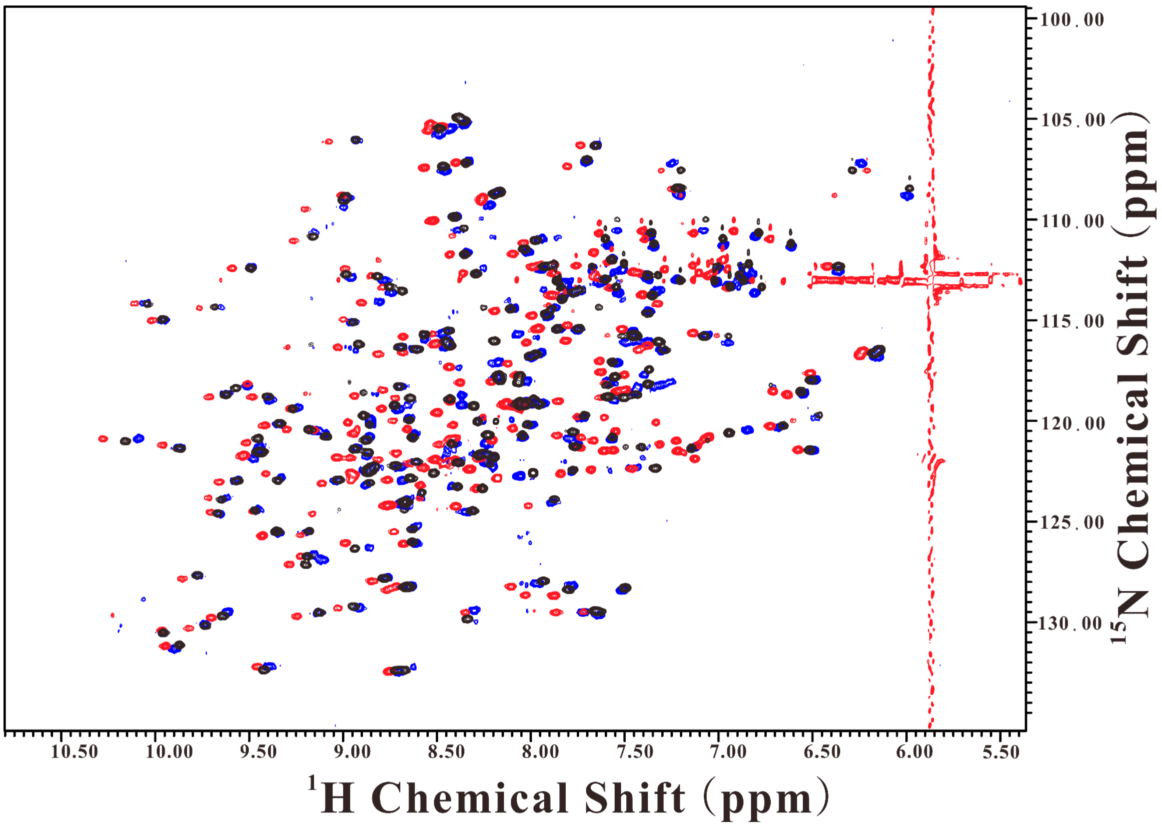

Figure S1. Two-dimensional ^{1}H-^{15}N-HSQC spectra of HGDC in (a) buffer, (b) 5.0 M urea, and (c) 1.0 M GdnHCl solutions. (d) An overlay of two-dimensional ^{1}H-^{15}N-HSQC spectra of HGDC in buffer (black), 5.0 M urea (red), and 1.0 M GdnHCl solutions (blue).

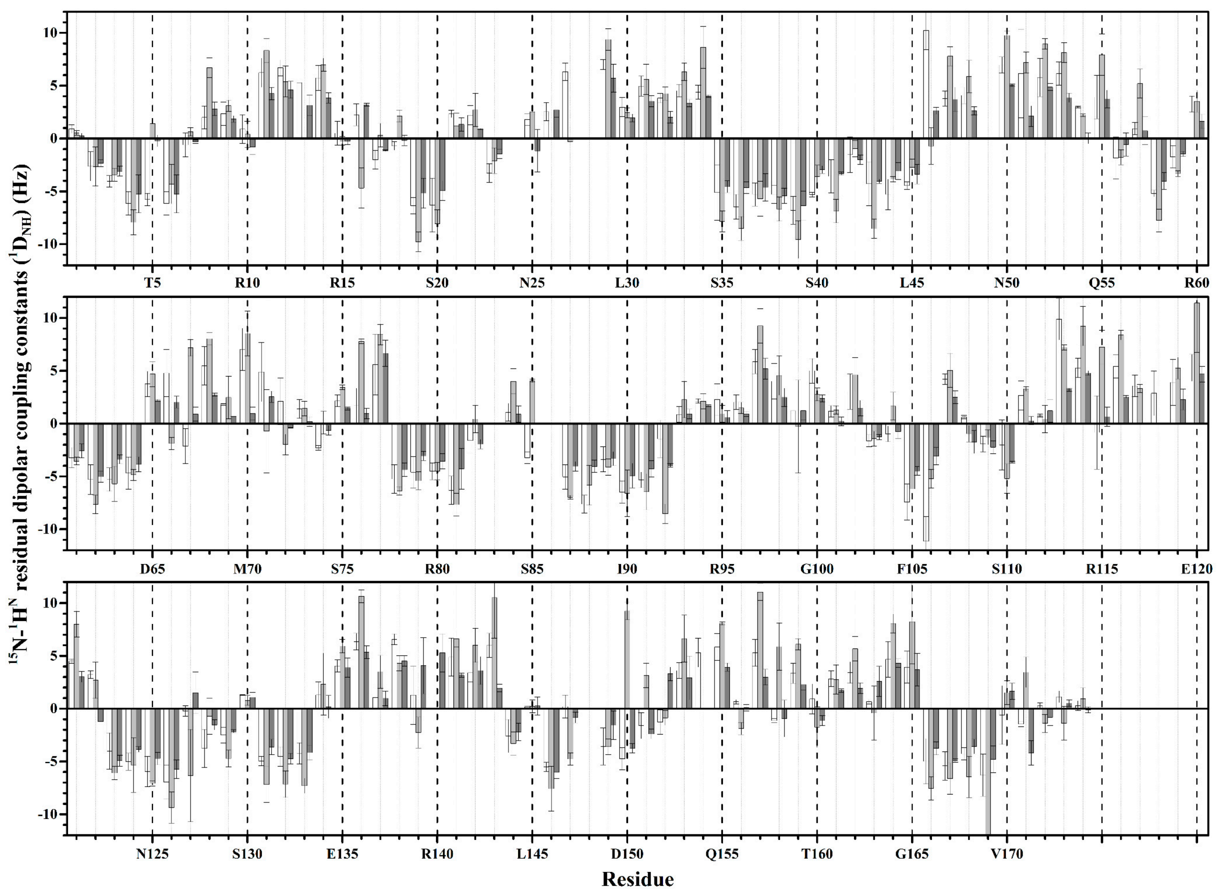

Figure S2. Experimental measured $^{1}D_{N\text{-}HN}$ RDCs of HGDC in buffer (white bar), 5.0 M urea (light gray bar), and 1.0 M GdnHCl (gray bar) solutions.

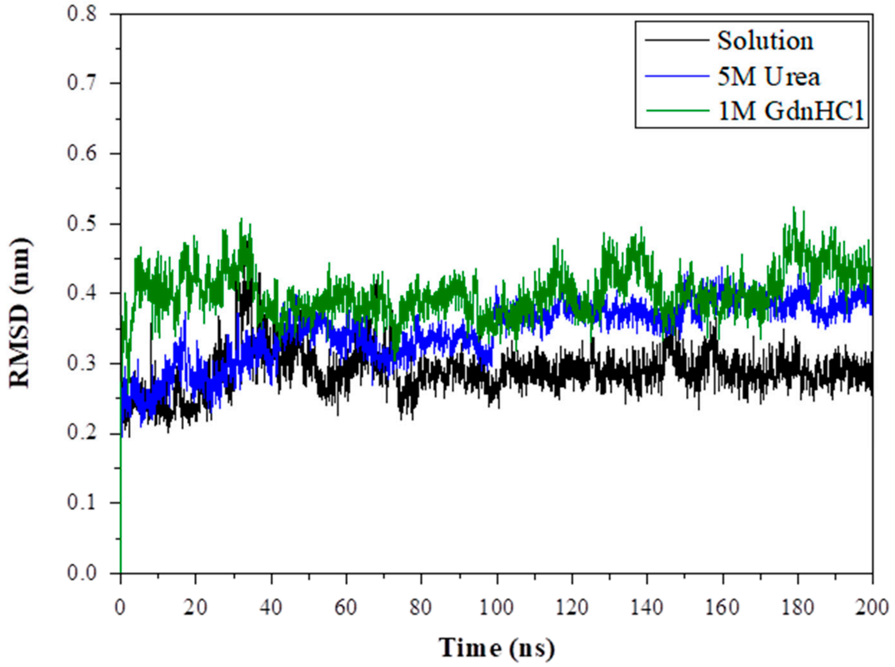

Figure S3. The backbone RMSD values of the HGDC NMR structures simulated in physiological solution (pH7, 136.7 mM NaCl), 343 K for 200 ns.

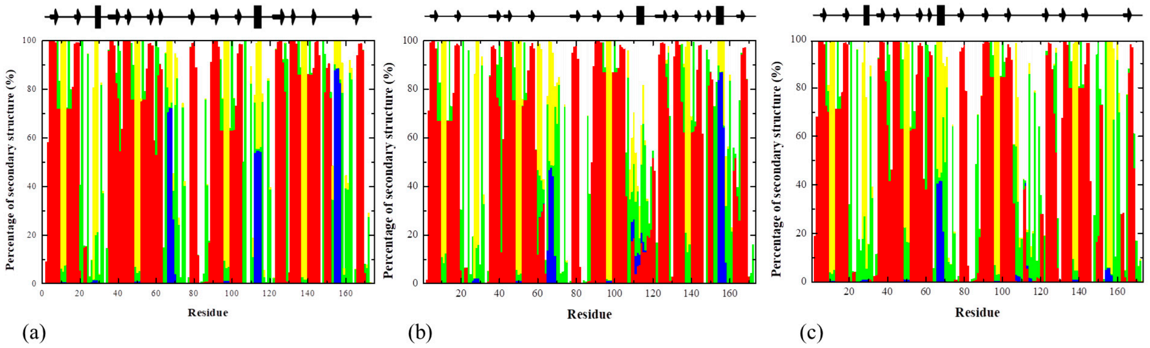

(a) (b) (c)

Figure S4. The percentage of secondary structures of the HGDC in physiological solution, 343 K, during 200 ns of simulation time for (a) solution, (b) Urea-induced, and (c) GdnHCl-induced structures. Red: β-strand; Blue: α-helix; Yellow: turn; Green: bend. The secondary structure cartoon of the initial conformer at the start of the simulation time for the respective structures is represented schematically above each graph in black with arrows and rectangles denoting β-strands and α-helices, respectively.

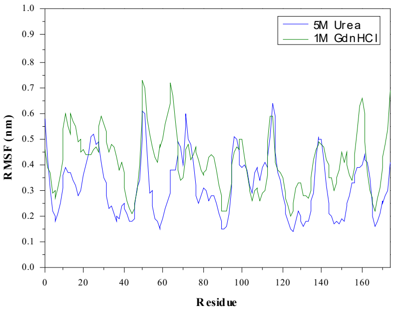

Figure S5. The backbone RMSF values for the higher energy state HGDC NMR structures induced by 5M urea and 1M GdnHCl. MD simulations were performed for 100 ns in physiological solution, 425K.

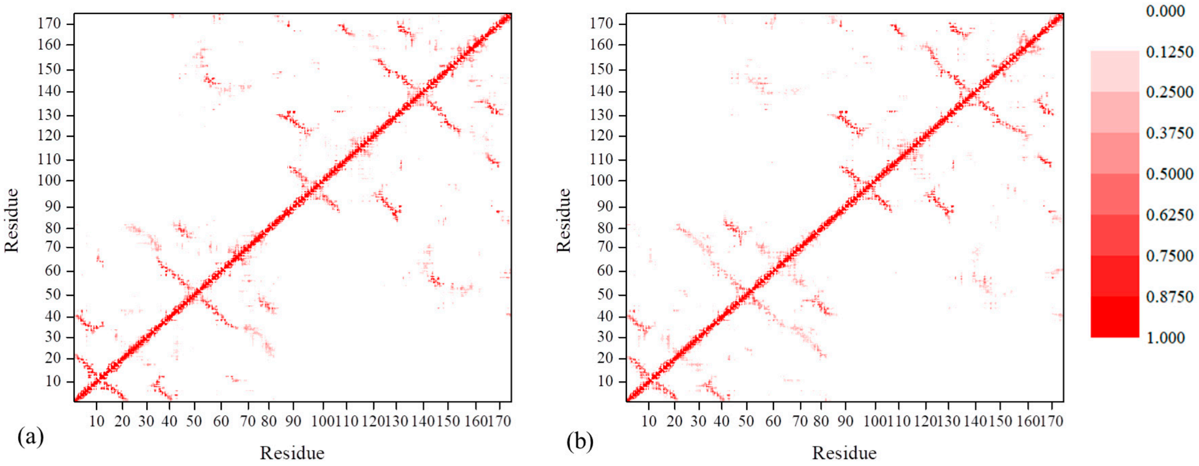

Figure S6. The 2D residue contact maps of (a) urea-induced and (b) GdnHCl-induced conformational ensembles throughout 100 ns of simulation time in physiological solution, 425K. Residue contacts of the initial conformation better maintained throughout the simulations are presented in darker red.

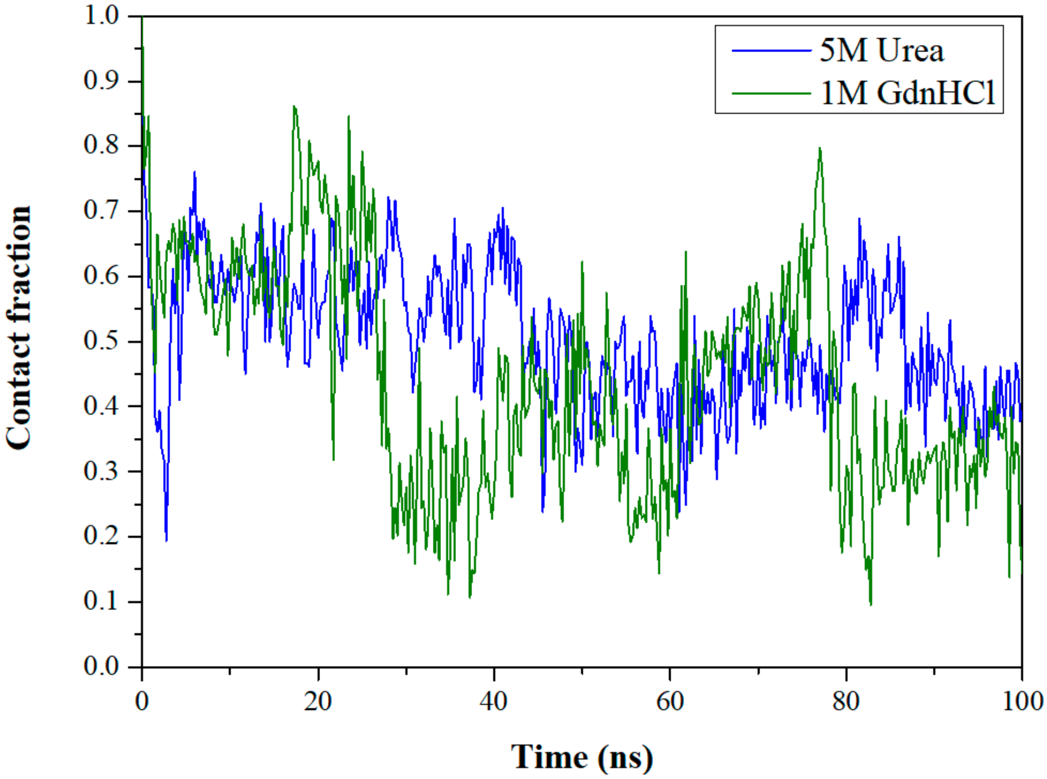

Figure S7. Inter-domain Interface contacts of HGDC throughout 100 ns simulation time in physiological solution, 425 K.
